# 969. Rehabilitation Therapies Not Limited by Concomitant Trauma in Large Total Body Surface Area Burns

**DOI:** 10.1093/jbcr/irag033.566

**Published:** 2026-04-06

**Authors:** Derek O Murray, Gabriella Viera, Julie M Meshanko, Karen J Richey, Claudia Islas, Chani Taggart, Gaby Iskander, Kevin N Foster

**Affiliations:** Diane & Bruce Halle Arizona Burn Center - Valleywise Health, Phoenix, AZ; Diane & Bruce Halle Arizona Burn Center - Valleywise Health, Phoenix, AZ; Diane & Bruce Halle Arizona Burn Center - Valleywise Health, Phoenix, AZ; Diane & Bruce Halle Arizona Burn Center - Valleywise Health, Phoenix, AZ; Valleywise Health Medical Center, Phoenix, AZ; Valleywise Health Medical Center, Phoenix, AZ; Valleywise Health Medical Center, Phoenix, AZ; Diane & Bruce Halle Arizona Burn Center - Valleywise Health, Phoenix, AZ

## Abstract

**Introduction:**

Many studies have examined medical and surgical management of the combined burn and trauma patient, but few have examined the provision of OT and PT in this specific population. The purpose of this study was to explore if trauma injury, when superimposed on large burns of similar size, resulted in additional limitations in the provision of inpatient OT and PT.

**Methods:**

This was a matched case-controlled retrospective chart review of patients treated at a verified burn center, who suffered concomitant burn and trauma versus burn injuries without trauma, with TBSA of at least 20%. Comprehensive chart review was performed. Data about therapy details of care for the burn group and the burn with combined trauma group was analyzed, on a per day length of stay and per day of therapy basis. Student t-tests were applied, using Python 3.11.

**Results:**

Forty subjects met criteria, 20 for each group. Data showed statistical significance in days to therapy evaluation, with therapy evaluations being completed sooner for the burn group (BG) than the burn and trauma group (BTG), (.89 days versus 1.73 days (*p*=.042). Patients in the BG were able to discharge directly home from the hospital significantly more often than in the BTG, (7 of 20 analyzed versus 1 of 20 (*p*=.017).There was no significant difference in any of the remaining data points, including all times to mobilization, missed therapy sessions or days per day LOS, the need for multiple skilled therapists simultaneously for treatments, or therapy days and minutes per LOS day between the BG and BTG.

**Conclusions:**

The burn therapy team was able to provide equivalent care to both groups once delay in initial evaluations in the BTG was overcome. Patients in the BG were more likely to DC home. Therapy services can be provided to patients with large TBSA burns and concurrent trauma injuries during the inpatient stay similarly to burn patients without concurrent trauma injuries.

**Applicability of Research to Practice:**

While concurrent trauma injuries delay initial therapy evaluations and alter discharge planning, they demonstrate no impact on the frequency, duration, or meeting key milestones for inpatient therapy rehabilitation.

**Funding for the study:**

N/A.

